# An Experimental Study on the Preparation of Soft Rock Similar Materials Using Redispersible Latex Powder as a Modifier

**DOI:** 10.3390/molecules27217404

**Published:** 2022-10-31

**Authors:** Xu Ren, Zhufang Zhang, Min Xiang, Guihong Xu, Wenze Cao

**Affiliations:** 1 College of Civil Engineering, Guizhou Institute of Technology, Guiyang 550003, China; 2 Guizhou Highway Engineering Group Co., Ltd., Guiyang 550025, China

**Keywords:** soft rock, similar materials, geomechanical model test, physical and mechanical indexes, plastic failure

## Abstract

The engineering geological problems of soft rock are common in large slope engineering and underground engineering surrounding rock. In order to study the change in mechanical properties of soft rock under the action of loading, excavation and rainfall, this paper carried out experimental research on similar materials of soft rock. The similar material of soft rock is prepared by using iron fine powder, barite powder and quartz sand as aggregate, gypsum as binder and redispersible latex powder as regulator. A single-factor influence test was designed with the content of redispersible latex powder as variation parameter. Analysis the influence of redispersible latex powder from the perspectives of physical and mechanical indexes, failure forms, stress–strain states and changes after water seepage. In addition, evaluate the feasibility of this similar material in geomechanical model test. Experimental results show that the density, compressive strength and Poisson’s ratio of similar materials can be improved to a certain extent by the redispersible latex powder with low dosage. However, the above indexes show a significant downward trend with the increase in dosage when the dosage exceeds 2%. The deformation modulus always shows a downward trend, and this trend becomes more significant especially when the dosage exceeds 2%. With the increase in the redispersible latex powder, the stress–strain curves of similar materials show obvious elastic and plastic stages. The failure mode gradually changes to X-shaped conjugate failure, which is common in soft rock, and the material changes from brittle failure to plastic failure. In addition, this type of similar material with gypsum as cementing agent will cause serious damage and loss of bearing capacity after seepage. These methods produce similar materials with low strength, low deformation modulus and plastic failure form, which can be used to simulate the stability of soft rock engineering caused by loading or excavation. At the same time, it also sheds lights on preparing similar materials of hard rock.

## 1. Introduction

**Figure 1:**
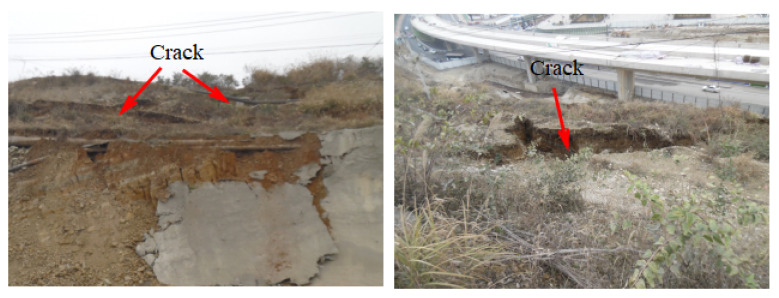
Drilling TV results ( engineering geology of soft rock).

In recent years, due to the increasingly frequent engineering activities and scale, the degree of disturbance to the geological environment has been aggravated, resulting in frequent large geological disasters because of the change in natural conditions in local areas. This is particularly common for the geological engineering problems of soft rock (Figure 1). A geomechanical model test is a common method to study complex engineering geological problems such as slope and underground engineering [1]. In order to ensure that geomechanical model test can truly reflect the relationship between geological structure and engineering construction, it is very important that the materials ( hereinafter referred to as similar materials) are similar to engineering rock, which determines the authenticity and validity of geomechanical model test results [2].

Scholars have carried out a series of laboratory experiments on similar materials for soft rock and obtained some results [3,4,5,6,7,8,9,10,11,12,13,14,15,16]. In the early stage of the study, quartz sand and barite powder were used as aggregate, and gypsum and cement were used as cementing agents to develop mixed materials satisfying the similar relationship in several aspects [3,4,5,6]. With the wide application of geomechanical model tests in engineering, the research of similar materials is also deepening gradually. For instance, Huang Kan, Li Yi and Yan Zhigang et al. [7] used quartz sand, kaolin, gypsum, laundry detergent and water to develop similar materials for the soft surrounding rock of tunnel grade V through the orthogonal test of “four factors and three levels”. It was concluded that the cohesion is improved, density decreased with the increase of kaolin. Chen Zhimin, Feng Yasong and Liu Dexue [8] used quartz sand, loess, cement, gypsum and tap water to develop similar materials for the surrounding rocks of grade IV, V and VI. It is found that cement can significantly improve the density, uniaxial compressive strength and cohesion of the material compared with gypsum. Therefore, researchers began to pay attention to the development of similar materials that meet certain specific similarity relations and special engineering geological conditions. For example, iron powder is added in order to increase the density [9,10], clay is added for low strength [7,8,9,10,11], ethylene glycol and hydraulic oil are added for simulate the rheological property of rock mass [9,10,11,12] and rubber powder is added for improve ductility [13]. The choice of materials is gradually diversified, and the requirements of various functions are gradually closer to the engineering reality.

From the current research, there are still the following problems in the development of similar soft rock materials:

First, the density of the developed materials is mostly lower than 2.0 g/cm${}^{3}$, which has a certain gap with the density of most soft rocks.

Second, the material in the premise of meeting the strength index often cannot obtain a matching deformation index.

Third, most studies [8,9,10,11,12,13,14,15,16] select materials and design mix ratios from some similar indexes, but ignore the similar relationship of failure characteristics and stress–strain state.

Finally, the stability of the slope and surrounding rock of underground structure is related to rainfall closely. However, few studies have considered the mechanical behavior and failure characteristics of similar materials after seepage.

Based on the above reasons, this paper will develop a material which is similar to soft rock in physical and mechanical indexes, failure mode, stress–state and so on. In this paper, quartz sand, barite powder, iron powder, gypsum and redispersible latex powder were used for mixing. Quartz sand, barite powder and iron powder are used as aggregate, gypsum as gelation material, redispersible latex powder (type: Vinyl Acetate-Ethylene, referred to as VAE) as modifier. VAE can increase the ductility, cohesion and water resistance. It is used in concrete commonly [17,18,19,20,21], which can play a role in regulating strength and deformation. Therefore, this paper designs a single-factor influence test with VAE dosage as the change parameter. The physical and mechanical indexes, stress–strain state and failure characteristics of the mixed material under different dosage were analyzed, and the applicability of VAE in a geomechanical model test of soft rock was evaluated. In addition, the change in the material after water seepage is analyzed, and the applicability of this type of material in the engineering stability of rain-eroded soft rock is discussed.

The conclusion is applied to the geomechanical model test of road slope engineering in the second phase of the Beijing East Road extension section in Guiyang City. In addition, this research results can also provide reference and basis for the development of similar materials for other soft rock geomechanical models, especially in large-scale model tests. Meanwhile, the similar materials of hard rocks are prepared by referring to VAE’s influence on the properties of similar materials of soft rocks.

## 2. Engineering Summary and Application Principle

### 2.1. Project Summary

**Figure 2:**
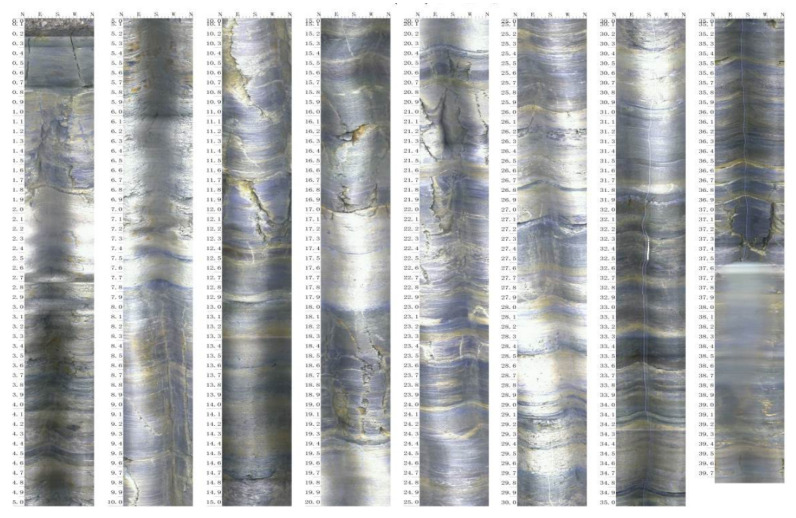
Drilling TV results (The yellow bands are mudstone intercalations).

The project is a second-phase road project of the extension section of the Beijing East Road in Guiyang City, Guizhou Province. The slope as a whole is a steep inner-bedding slope. The strata occurrence interval is 200${}^{\circ }$∼400${}^{\circ }$ ∠ 11${}^{\circ }$∼30${}^{\circ }$. The field area belongs to the Yangtze platform→Qianbei platform→Zunyi broken arch→Guiyang complex structural deformation zone. According to the geological mapping, no fault is found in the field area. The whole rock layer is monocline, and the interlayer contains a mud interlayer. The structural plane is flat and smooth, and the local joint is very poor, which is a weak structural plane. Figure 2 is the borehole TV scan result obtained by drilling sampling. The yellow band in the figure is the mudstone interlayer, and the rest is argillaceous limestone. Therefore, it can be concluded that the rock mass combination of the slope area is argillaceous limestone and mudstone, and the thickness of the interlayer mudstone is about 5–30 cm, accounting for about 8% of the total layer thickness. Among them, the joint fissures of rock are developed. The local mudstone layer is weathered seriously, which has mud phenomena.

### 2.2. The Similarity Theory of Model Test

As the basis of physical model tests, the similarity theory can be correctly applied to ensure that the strength and deformation law of rock mass can be truly restored by model tests [22]. Here, *C* is defined as the similarity ratio between the physical quantities of prototype (*p*) and model (*m*):

(1) $$C_{i}=q_{ip}/q_{im}$$

$q_{ip},q_{im}$—One physical quantity in a prototype (*p*) or model (*m*).

Through dimensional analysis, the three basic equations of geometry, physics and equilibrium can be deduced based on boundary conditions as follows:

(2) $$C_{\epsilon }C_{E}C_{l}^{-1}=C_{\rho }C_{g}$$

ϵ—strain (Dimension is 1);

*l*—length (m);

ρ—density (g/cm${}^{3}$);

*E*—deformation modulus (MPa);

*g*—gravity acceleration (m/s${}^{2}$).

In the model test, we try to ensure that the strain and gravity acceleration are not distorted, so that $C_{g}=1,C_{\epsilon }=1$.

Expression (2) can be obtained:

(3) $$C_{E}=C_{\rho }C_{l}$$

Taking the strain similarity proportion, length similarity proportion and density similarity proportion as the basic unknowns, it can be deduced that the stress similarity proportion needs to meet the following conditions:

(4) $$C_{\sigma }=C_{E}C_{\epsilon }=C_{\rho }C_{l}C_{\epsilon }$$

σ—Stress (MPa).

### 2.3. Physical Mechanical Parameter

By collecting 16 samples, the physical and mechanical parameters of mudstone in natural state were obtained. In addition, in order to facilitate the geomechanical model test under the gravity stress field, the gravity acceleration and density similarity ratio is adopted as $C_{g}=1$, $C_{\rho }=1$ in the preparation process of similar materials, which can greatly reduce the conversion work between the model materials and the physical and mechanical characteristic parameters of the actual engineering rock mass [23,24]. Considering the maneuverability of the large-scale slope engineering model test, the geometric similarity ratio was determined to be $C_{l}=100$. According to the principle of similarity, the characteristic parameters of physical and mechanical indexes of similar materials are determined. The physical and mechanical indexes of mudstone (natural state) and similar materials are shown in Table 1.

## 3. Test Process

### 3.1. Preliminary Preparation

**Figure 3:**
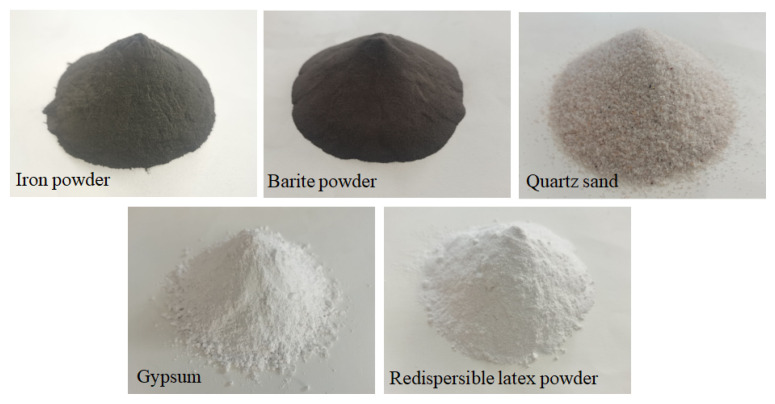
Actual photos of the raw materials.

In order to develop the material with similar density index and determine the curing time of the material, the preliminary preparation was carried out first. On the basis of previous research experience [25,26,27,28,29,30], the selected materials are as follows: iron powder (fine aggregate, 150 mesh, density ∼5.12 g/cm${}^{3}$); barite powder (fine aggregate, 100 mesh, density ∼4.2 g/cm${}^{3}$); and quartz sand (coarse aggregate, 16–26 mesh, density ∼2.65 g/cm${}^{3}$). Gypsum was used as the cementitious material. Figure 3 is the actual photos of the raw materials. The preliminary designed mixing ratio is as follows: gypsum powder:water:iron essence:barite powder:quartz sand = 1.0:1.60:1.35:6.08:3.85. Four groups of specimens were prepared, and all solid materials were added to the stirrer in accordance with the above ratio and dry mixed for 30 s. After mixing the solid materials evenly, add water and mix for 90 s. Next, this material is loaded into the mold (70 mm cube mold) and compacted. Static placing for 2 h at room temperature before mold release. They were, respectively, cured for 3 days, 5 days, 7 days and 12 days in an indoor dry environment, without adding water. After curing, the density, uniaxial compressive strength and deformation index of the specimen were tested. The density was calculated by weighing the mass of the specimen to an accuracy of 0.001 g. The compressive strength and deformation modulus are tested on a 10KN microcomputer-controlled universal testing machine to control the displacement speed of loading. The speed is 0.05 mm/min. The test results are shown in Table 2.

**Figure 4:**
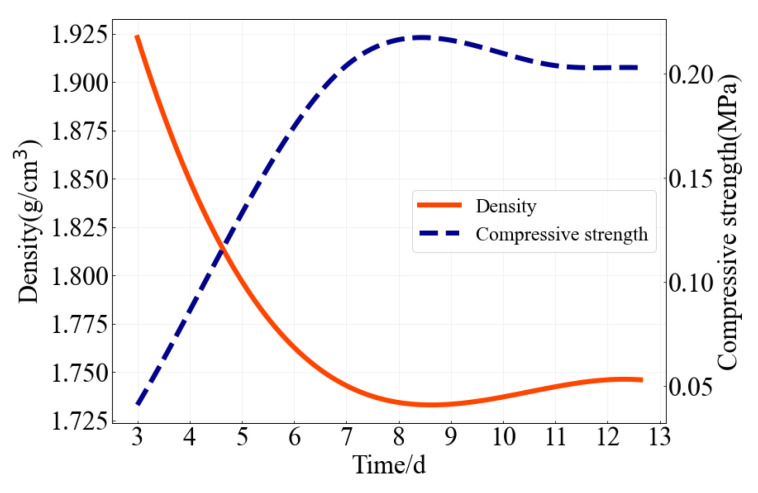
Relation curve between density, compressive strength and curing time of similar materials. Note: The variation law of deformation modulus is basically the same as that of compressive strength.

As can be seen from the test results in Table 2 and Figure 4, the density is smaller than most of those soft rocks [31,32,33]. Therefore, the proportion of iron concentrate powder and barite powder in the total aggregate and the proportion of iron concentrate powder in fine aggregate can be appropriately increased to meet the density requirements. In addition, at the end of 7 days of curing, each index value basically remained unchanged. Therefore, the type of similar materials with gypsum as cementing material can be tested after curing for 7 days. However, the rate of water evaporation is different because the size of the test is different. Therefore, the specific curing time can be determined by combining with the density index. In other words, when the density is constant, the curing is ended.

### 3.2. Specimen Production of Similar Material

On the basis of the above experimental results, VAE (packing density: 1 g/cm${}^{3}$) was incorporated into the mixed material as a regulator. The proportion of VAE is used to design the test with single-factor influence. The specific ratio is shown in Table 3.

**Figure 5:**
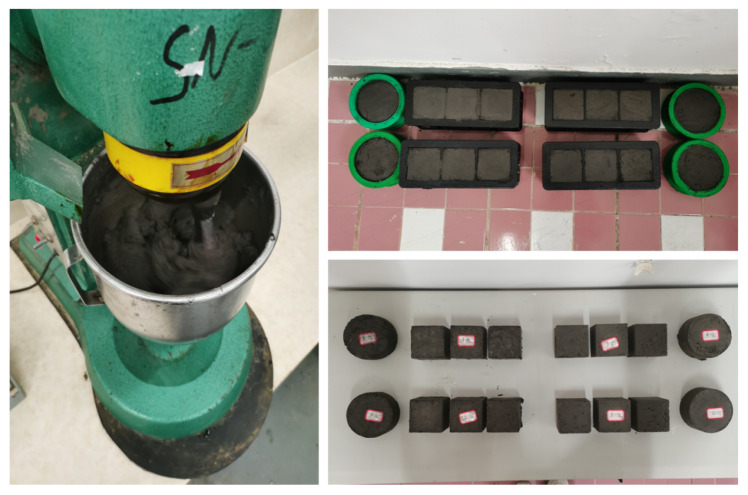
Preparation and curing of specimens.

(1) Raw material weighing: According to the test mix ratio in Table 3, the dosage of various materials was weighed, accurate to 0.1 g.

(2) Stirring: The aggregate, cementitious material and VAE are added to the mixing bucket in turn. After stirring for 30 s, water was poured in and continuously stirred for 90 s.

(3) Putting into the mold: Putting the mixed material into a 70 mm × 70 mm × 70 mm cube mold and vibrating until it was tight.

(4) Demoulding and maintenance: Under normal temperature conditions, demoulding and numbering was performed after 2 h. According to the above conclusion, the specimens should be cured in a dry room for at least 7 days, and the weight of the specimens should remain unchanged. Figure 5 is the specimen in the process of preparation and curing.

It was found that with the increase in VAE dosage, the fluidity of the mixture increased significantly. The water retention of the mixture was enhanced after the test. It can be speculated that VAE can improve the performance of similar materials and is conducive to the simulation of soft rock [31,32,33] formations strata with different thicknesses. It is convenient to carry out the geomechanical model test.

## 4. Test and Analysis of Results

### 4.1. Experimental Testing

**Figure 6:**
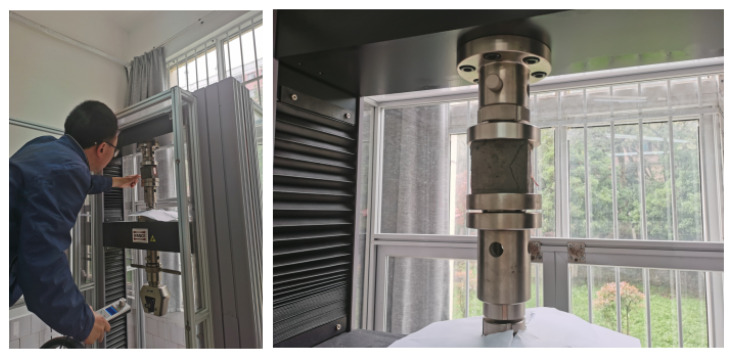
Test of similar materials.

After curing, the density, uniaxial compressive strength and deformation modulus of each group of specimens were tested, and the test process is shown in Figure 6.

**Figure 7:**
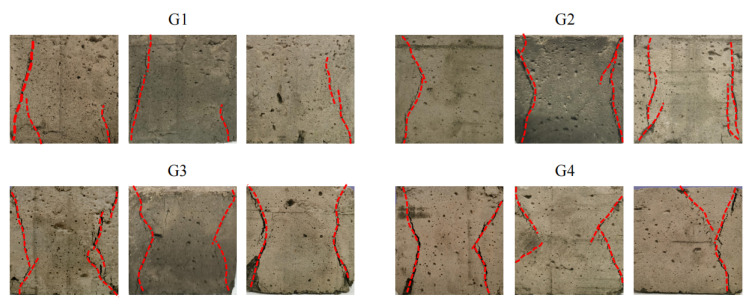
Macro-rupture characteristics of similar materials.

**Figure 8:**
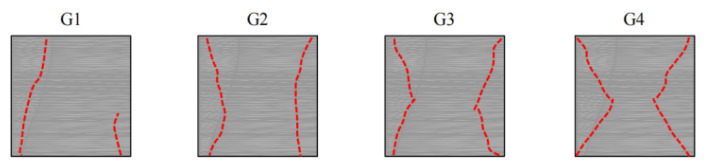
Diagram of similar material damage characteristics.

By observing Figure 7 and Figure 8, it can be found that when VAE is not added, the material exhibits tensile failure caused by transverse tensile stress due to Poisson effect. However, with the increase in VAE, the damage characteristic changes from tensile failure to X-shaped conjugate failure [34,35,36,37], which is common in soft rock. It can be inferred that when VAE is not added, brittle failure forms [35,36] are the same as most of those hard rocks, and its macro failure characteristics are not consistent with those of soft rocks. The addition of VAE improves the tensile strength of the material, resulting in the transformation of the failure mode into plastic failure, which is further proved in the following stress–strain state analysis.

### 4.2. Analysis of Test Results

**Figure 9:**
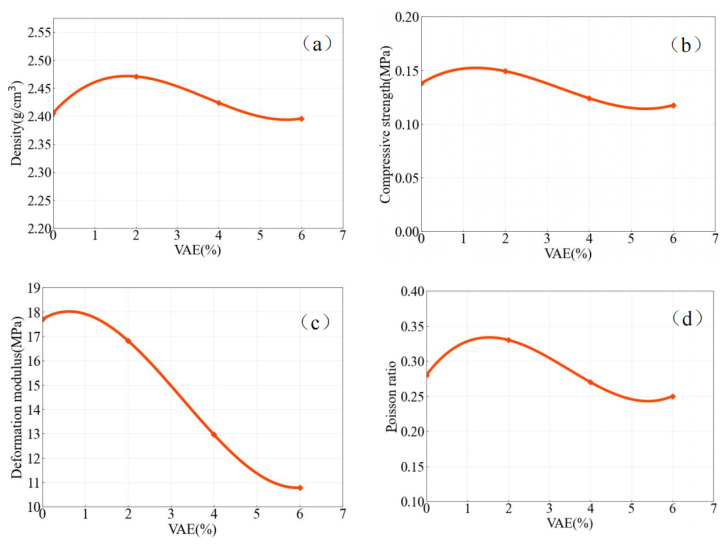
Relationship between VAE and indexes with different dosage. (**a**) Density. (**b**) Compressive strength. (**c**) Deformation modulus. (**d**) Poisson ratio.

Table 4 Physical and mechanical indicators of similar materials:

After the test, the physical and mechanical indicators are shown in Table 4. Figure 9a–d shows the relationship between density, compressive strength, deformation modulus, Poisson ratio and the dosage of VAE.

From Figure 9a, it can be seen that the density shows an obviously increasing trend when the dosage of VAE is less than 2% compared with that without VAE. It can be speculated that a small amount of VAE can increase the degree of aggregation between the components, thereby improving the compactness of similar materials, resulting in an increase in density. When the dosage exceeds 2%, the density shows a downward trend. At this time, the aggregation effect of VAE can still increase the density of similar materials to a certain extent, but this effect is obviously weakened with the further increase in VAE. When the dosage exceeds 6%, the density is significantly lower than that when the dosage is 0%. Due to the low density of VAE, when the dosage increases to a certain level, it must play a dominant role in reducing the density of similar materials.

In addition, the compressive strength (Figure 9b) increased first and then decreased with the increase in VAE. When the dosage is less than 2%, the aggregation effect of VAE significantly promotes compressive strength. When the dosage exceeds 2%, the compressive strength of similar materials decreases sharply. It can be seen that the strength increase caused by the aggregation of VAE has been gradually replaced by its weakening effect on strength. The Poisson ratio (Figure 9d) has a similar variation law with compressive strength. In addition, the deformation modulus (Figure 9c) decreased nonlinearly with the increase in VAE, and the initial deceleration was not obvious. When VAE exceeds 2%, the deformation modulus decreases significantly with the increase of VAE.

### 4.3. Similarity of Stress–Strain State

**Figure 10:**
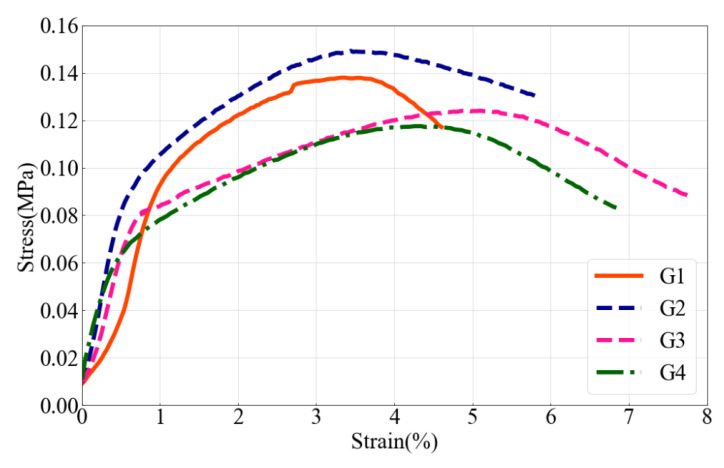
Stress–strain curves of similar materials.

Figure 10 shows the stress–strain curves of similar materials in each group. It can be found that with the increase in VAE, the stress–strain curves gradually show an obvious inflection point. Particularly, when the VAE exceeds 4%, the distinction between elastic and plastic stages is more pronounced, which is consistent with the properties exhibited by the macroscopic fracture morphology of similar materials. It is concluded that with the increase in VAE, the ductility of similar materials is enhanced, and the failure mode changes from brittle failure to plastic failure, which is similar to most soft rock failure modes [35,36,38,39,40].

### 4.4. Similar Material after Water Seepage

The four groups of specimens were infiltrated with water at a slow flow rate until the specimens were saturated with water, which took about 45 min. The saturated water absorption rate of the four groups of specimens was about 9%.

**Figure 11:**
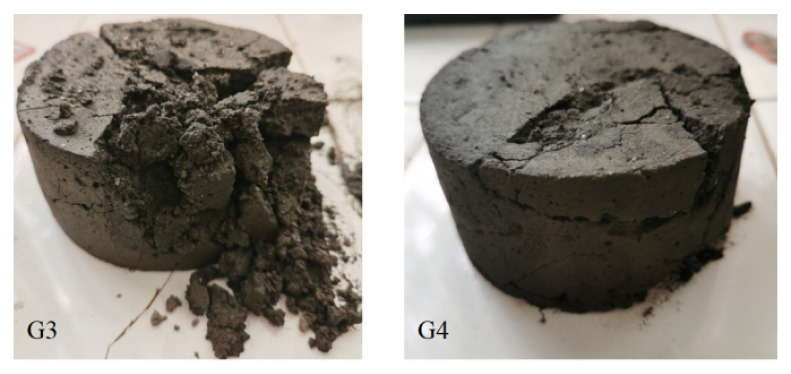
Failure modes of G3 and G4 specimens after water percolation.

The bonding between the solid particles of the G1 and G2 specimens almost disappeared, resulting in overall collapse. In addition, by weighing the mass of the G3 and G4 specimens, it was concluded that the mass loss rates of the G3 and G4 specimens are about 45.0% and 17.5%, respectively. Figure 11 shows the failure modes of G3 and G4, which have softened and lost their bearing capacity. In general, it can be seen that the incorporation of VAE is beneficial to improve the integrity of the material after water seepage. The dihydrate gypsum crystals will dissolve in water and cause the overall collapse of the material, which still plays the main control role. Additionally, this kind of failure is obviously different from the failure form of most soft rocks [35,36,41,42,43] after the infiltration of rainwater. In view of the above failure phenomenon, the mechanical behavior of the hybrid material is not tested in this paper.

### 4.5. Discussion of Test Results

In this paper, similar materials of soft rock have been developed. Next, the advantages and problems of this kind of similar materials are discussed from the following three aspects.

(1) The experimental results show that the compressive strength of this material is less than 0.1492 MPa and the deformation model 17.69 MPa. Therefore, it is more beneficial to be used as a substitute material in large-scale model tests of soft rock engineering, while it has limitations in small-scale model tests of hard rock or soft rock engineering.

(2) The physical and mechanical properties of similar materials of this type can be adjusted according to the actual requirements, which is more advantageous in simulating the stability problems of soft rock engineering due to pile loading or excavation. However, it is worth noting that the failure form is different from that of soft rock due to the collapse after meeting water. Therefore, this material cannot be used to analyze the stability of soft rock engineering caused by rainfall.

(3) Most researchers use VAE as a modifier for concrete. The application and research in geomechanical model tests are rare. On the premise that the density is not less than 2.4 g/cm${}^{3}$, 2∼4% VAE can significantly reduce the strength and deformation modulus. Failure morphology and stress–strain state can also reduce soft rock to a certain extent. Therefore, compared with other commonly used regulators, VAE can reduce the actual mechanical behavior of soft rock more truly and effectively.

## 5. Application of Research Results

Combined with engineering practices, similar materials of mudstone were prepared according to the physical and mechanical characteristic parameters of similar materials, as shown in Table 1. In view of the above test and result analysis, the same mixed material is selected, and the content of VAE is 3%. The physical and mechanical characteristic parameters of similar materials are shown in Table 5. In addition, because the material failure form is X-shaped conjugate failure, and the stress–strain state presents an obvious elastic–plastic stage, the material belongs to plastic failure, which is similar to the failure form of mudstone. This material can be applied to the geomechanical model test of the above-mentioned slope engineering.

## 6. Results and Discussion

(1) The experiment used iron powder, barite powder, quartz sand, gypsum, dispersible polymer powder and water as raw materials to develop similar materials for soft rock. Compared with the existing research results, this paper adds to the discussion on the similarity relationship of failure form, stress–strain state and changes after water seepage, and analyzes the similarity of this materials from different angles. The conclusions can be used to simulate the stability of soft rock in large-scale slope engineering or surrounding rock engineering of underground structures due to piling or excavation.

(2) Similar materials that use gypsum powder as a cementing material to prepare soft rock generally need to be cured in a dry indoor environment for at least 7 days or to a constant weight. When the dosage of VAE increased from 0% to 6%, the material gradually showed X-shaped conjugated destruction obviously, which transitions from brittleness to ductility. This ductility failure achieves macroscopic failure characteristics similar to those of soft rock.

(3) When the dosage of dispersible polymer powder is within 2%, the density, compressive strength and Poisson ratio of similar materials all show an upward trend with the increase in dosage. After more than 2%, the aforementioned indicators and deformation modulus have a significant downward trend. Compared with the unincorporated dispersible polymer powder, the compressive strength decreased by a maximum of 15% and the deformation modulus 39%. However, this influence degree is related to the selected aggregate and gelling agent, and this paper does not make a comprehensive evaluation. Therefore, authors will compare other types of mixed materials to further explore the effects of VAE on the performance of similar materials.

(4) Generally speaking, when the VAE dosage is controlled in the range of 2∼4%, the physical and mechanical indexes, failure forms and stress–strain state of materials play an obvious benign regulating role. This regulating role can also be applied to the development of similar materials for hard rock.

(5) The mass loss rate of this similar material is not less than 17.5% due to dissolution after meeting water, and the bearing capacity is basically lost, which is quite different from that of soft rock. It is not suitable to analyze the stability of soft rock engineering caused by rainfall, so it is still defective in application.

(6) In the preparation of soft rock similar materials, most studies discuss the similarity problem after the material is fully cured. In fact, the curing time can be adjusted according to actual needs. That is to say, when the strength develops to a suitable degree, the curing will end. For example, if cement is used as a binder, similar problems of these materials can be analyzed on the basis of curing within 28 days. In addition, clay or heavy calcium carbonate powder can also be considered to adjust the physical and mechanical properties of similar materials.

## Figures and Tables

**Table 1:** Physical and mechanical indexes of mudstone and similar materials.

	Density (g/cm${}^{3}$)	Compressive Strength (MPa)	Deformation Modulus (MPa)	Poisson Ratio
mudstone	2.41∼2.63	10.0–25.5	1300–3100	0.23∼0.31
similar materials of mudstone	2.41∼2.63	0.10–0.26	13–31	0.23∼0.31

**Table 2:** Physical and mechanical indexes of similar materials from primary tests.

Curing Day	Density (g/cm${}^{3}$)	Compressive Strength (MPa)	Deformation Modulus (MPa)
3 days	1.923	0.041	4.430
5 days	1.797	0.133	28.515
7 days	1.743	0.204	42.562
12 days	1.746	0.203	41.813

**Table 3:** Mixture ratio of similar materials.

Group	Ferric Powder	Barite Powder	Quartz Sand	Gypsum	Water	VAE
G1	6.67	1.33	2.22	1	1.56	0%
G2	6.67	1.33	2.22	1	1.56	2%
G3	6.67	1.33	2.22	1	1.56	4%
G4	6.67	1.33	2.22	1	1.56	6%

**Table 4:** Physical and mechanical indexes of similar materials with different VEA dosage.

Group	Density (g/cm${}^{3}$)	Compressive Strength (MPa)	Deformation Modulus (MPa)	Poisson Ratio
G1 (0%)	2.406	0.1378	17.69	0.28
G2 (2%)	2.471	0.1492	16.81	0.33
G3 (4%)	2.424	0.1240	12.96	0.27
G4 (6%)	2.396	0.1175	10.78	0.25

**Table 5:** Physical and mechanical indexes of similar materials for mudstone.

	Density (g/cm${}^{3}$)	Compressive Strength (MPa)	Deformation Modulus (MPa)	Poisson Ratio
Similar materials of mudstone	2.44	0.1355	14.21	0.28

## Data Availability

The data presented in this study are available on request from the corresponding author.

## Sample Availability

Samples of the compounds prepared in this article are available from the authors.
